# Möglichkeiten der künstlichen Intelligenz im Strahlenschutz: Verbesserung der Sicherheit bei medizinischen Bildgebungsuntersuchungen

**DOI:** 10.1007/s00117-023-01167-y

**Published:** 2023-06-22

**Authors:** Ali Pashazadeh, Christoph Hoeschen

**Affiliations:** Institut für Medizintechnik (IMT), Otto-von-Guericke-Universität Magdeburg, Otto-Hahn-Str. 2, 39016 Magdeburg, Deutschland

**Keywords:** Medizinische Bildgebung, Reduzierung der Strahlendosis, Computertomographie, Angiographie, Durchleuchtung, Medical imaging, Reduced radiation dose, Computed tomography, Angiography, Fluoroscopy

## Abstract

**Klinisch-methodisches Problem:**

Die Bildgebung von Strukturen im Körperinneren erfordert oft den Einsatz ionisierender Strahlung, die grundsätzlich ein Gesundheitsrisiko darstellt. Eine Reduktion der Strahlendosis kann zu verrauschten Bildern führen, die klinisch weniger aussagekräftig sind.

**Radiologische Standardmethoden:**

Das Problem tritt bei häufig verwendeten medizinischen Bildgebungsmodalitäten wie Computertomographie (CT), Positronen-Emissions-Tomographie (PET), Einzelphotonen-Emissions-Computertomographie (SPECT), Angiographie, Fluoroskopie und allen Modalitäten auf, bei denen ionisierende Strahlung zur Bildgebung eingesetzt wird.

**Methodische Innovationen:**

Die künstliche Intelligenz (KI) könnte die Qualität von Niedrigdosisbildern verbessern und dazu beitragen, die erforderliche Strahlung zu minimieren. Mögliche Anwendungen werden untersucht, und es erfolgt eine kritische Bewertung der Rahmenbedingungen und Verfahren.

**Bewertung:**

Die Leistung der KI-Modelle variiert. Hochleistungsmodelle könnten in naher Zukunft im klinischen Umfeld eingesetzt werden. Für eine optimale Leistung und eine breite Einführung dieser Technologie in der medizinischen Bildgebung müssen noch einige Herausforderungen (quantitative Genauigkeit, unzureichende Trainingsdaten etc.) angegangen werden.

**Praktische Empfehlungen:**

Um das Potenzial von KI und Deep Learning (DL) in der medizinischen Bildgebung voll auszuschöpfen, müssen Forschung und Entwicklung intensiviert werden. Insbesondere muss die Qualitätskontrolle der KI-Modelle sichergestellt werden, und die Trainings- und Testdaten müssen unkorreliert und qualitätsgesichert sein. Bei hinreichender wissenschaftlicher Absicherung und rigorosem Qualitätsmanagement könnte die KI zu einem sicheren Einsatz von Niedrigdosistechniken in der medizinischen Bildgebung beitragen.

## Abstract (en)

**Clinical/methodological issue:**

Imaging of structures of internal organs often requires ionizing radiation, which is a health risk. Reducing the radiation dose can increase the image noise, which means that images provide less information.

**Standard radiological methods:**

This problem is observed in commonly used medical imaging modalities such as computed tomography (CT), positron emission tomography (PET), single photon emission computed tomography (SPECT), angiography, fluoroscopy, and any modality that uses ionizing radiation for imaging.

**Methodological innovations:**

Artificial intelligence (AI) can improve the quality of low-dose images and help minimize radiation exposure. Potential applications are explored, and frameworks and procedures are critically evaluated.

**Performance:**

The performance of AI models varies. High-performance models could be used in clinical settings in the near future. Several challenges (e.g., quantitative accuracy, insufficient training data) must be addressed for optimal performance and widespread adoption of this technology in the field of medical imaging.

**Practical recommendations:**

To fully realize the potential of AI and deep learning (DL) in medical imaging, research and development must be intensified. In particular, quality control of AI models must be ensured, and training and testing data must be uncorrelated and quality assured. With sufficient scientific validation and rigorous quality management, AI could contribute to the safe use of low-dose techniques in medical imaging.

## Hintergrund

Künstliche Intelligenz (KI) erlaubt vielversprechende Veränderungen in der Medizin und der medizinischen Bildgebung, indem sie Aufgaben wie Erkennung, Klassifizierung, Charakterisierung, Überwachung, Radiomics, strukturierte Berichterstattung und Arbeitsabläufe verbessert. KI ermöglicht die Analyse medizinischer Bilder mit hoher Genauigkeit, wodurch die Wahrscheinlichkeit falsch-positiver und -negativer Erkennungen verringert wird. Durch die Kategorisierung medizinischer Befunde ermöglicht die KI eine bessere Risikostratifizierung und potenziell eine personalisierte Versorgung der Patienten. Bei der Charakterisierung/Segmentierung medizinischer Bilder kann KI helfen, die Grenzen, z. B. von Läsionen, genau zu bestimmen. Der Einsatz von KI-Technologie kann auch eine effiziente Verlaufskontrolle ermöglichen, z. B. bei der Beurteilung der Tumorresponse auf eine Therapie. KI kann in Radiomics wirksam eingesetzt werden, um quantitative Merkmale zu extrahieren und zu analysieren und sie mit anderen Datentypen zu korrelieren, oder um umfassende Patientensignaturen für die personalisierte Behandlungsplanung zu erstellen. Ein strukturiertes, von KI unterstütztes Reporting kann die Sammlung und Speicherung von Informationen über Einrichtungen und Studien hinweg standardisieren und die Erstellung patientenbezogener Berichte erleichtern. Auch zur Optimierung von Arbeitsabläufen kann die KI beitragen, ebenso zur Optimierung der Arbeitsabläufe, der Terminplanung sowie der Bilderfassung zur Verbesserung der Gesamteffizienz. Darüber hinaus kann KI die Befundung beschleunigen und mühsame Aufgaben wie die Detektion und Quantifizierung von Herdbefunden automatisieren.

In Zusammenhang mit der Bildrekonstruktion kann die KI zur Verbesserung der Bildqualität beitragen, die bei niedrigen Strahlendosen durch Rauschen und weitere Artefakte beeinträchtigt ist oder sein kann. Eine Reduktion der Strahlendosis bedeutet bei der Transmissionsbildgebung wie der Röntgenuntersuchung oder Computertomographie (CT) eine Reduzierung des Photonenflusses (durch Reduktion von kVp und/oder mAs), bei der Emissionsbildgebung wie Szintigraphie oder Positronen-Emissions-Tomographie (PET) eine Reduzierung der Aktivität der applizierten Tracer. Sie hat eine Minderung des Signal-Rausch-Verhältnisses (SNR) und damit eine Verschlechterung der Bildqualität zur Folge – mittelbar eine verminderte klinische Aussagekraft der berechneten Bilder. Verschiedene Algorithmen zur Bildentrauschung sind bisher evaluiert worden, und ihre Leistungsfähigkeit zeigt sich als begrenzt. So erzeugen beispielsweise herkömmliche Bildrekonstruktionsverfahren in der Regel suboptimale und fleckige Bilder, wenn sie übermäßig auf Niedrigdosis-CT-Bilder angewandt werden, was ihre Verwendbarkeit für diagnostische Zwecke einschränkt [1].

**Figure 1:**
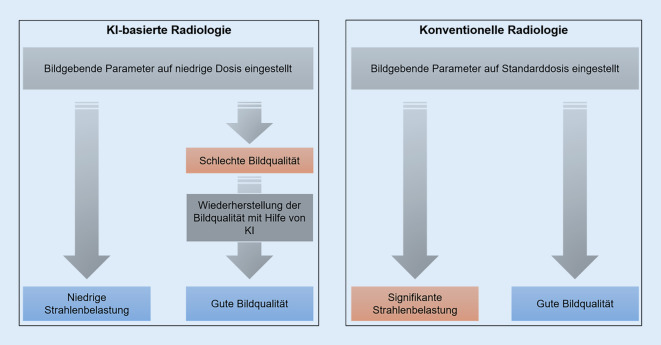

KI-basierte Entrauschungsalgorithmen hingegen können unter bestimmten Rahmenbedingungen durch die Zuordnung von medizinischen Niedrigdosisbildern zu ihren Normaldosisbildern das Signal in den verrauschten Strukturen wirksam wiederherstellen. Ein KI-basierter Algorithmus kann entweder Rohdaten, die mit niedriger Dosis aufgenommen wurden, vor der Rekonstruktion entrauschen oder Rauschen im Bildbereich entfernen, oder sogar lernen, qualitativ hochwertige Bilder von Grund auf zu rekonstruieren, was zu schnelleren und effizienteren Ergebnissen führen kann als herkömmliche Rekonstruktionstechniken. Durch die Ergänzung unvollständiger Datensätze, die mit Hilfe der Niedrigdosisbildgebung gesammelt wurden, reduziert die KI die Strahlenexposition bei potenziell ähnlicher Bildqualität. Darüber hinaus kann KI, ausgehend von nichtionisierenden Bildgebungstechniken wie der Magnetresonanztomographie (MRT), auch Pseudo-CT-Bilder erstellen und so die ionisierende Bildgebung in den Fällen ersetzen, in denen dies aus klinischer Sicht möglich ist. Die Integration der KI-Technologie in herkömmliche radiologische Arbeitsabläufe für einen sichereren und effektiveren Bildgebungsprozess ist in Abb. 1 dargestellt.

Im Folgenden werden einige Studien besprochen, in denen KI-Algorithmen entwickelt und eingesetzt wurden, um Bilder zu entrauschen, die unter Niedrigdosisbedingungen aufgenommen wurden, z. B. bei niedrigem kVp, niedrigem mAs oder niedriger Aktivität, und ihre Qualität so zu verbessern, dass sie dem Standard von Normaldosisbildern entspricht. Der Inhalt ist in 5 Abschnitte gegliedert, die den häufig verwendeten Bildgebungsmodalitäten CT, PET, Einzelphotonen-Emissions-Computertomographie (SPECT), Angiographie und Fluoroskopie entsprechen.

## Künstliche Intelligenz in der Niedrigdosis-CT

**Figure 2:**
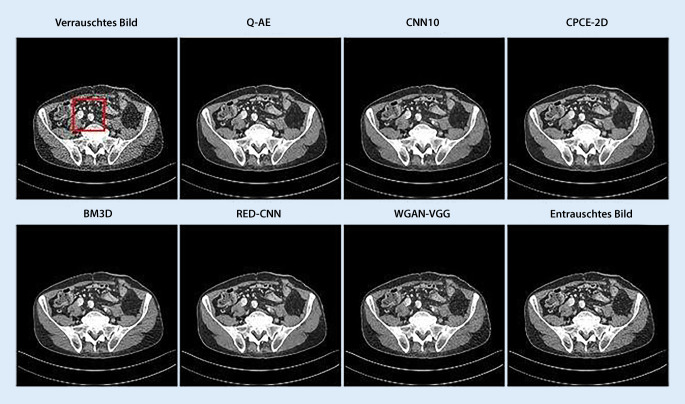

Wie verschiedene KI-basierte Methoden das Rauschen von Niedrigdosis-CT-Bildern reduzieren und ihre Lesbarkeit verbessern können, zeigt Abb. 2. Die für das Entrauschen von Niedrigdosis-CT-Bildern entwickelten KI-Modelle lassen sich im Allgemeinen in 3 Hauptmodelle einteilen:

- diskriminativ, d. h. eine Bottom-up-Ausführung zur Trennung der Daten anhand einer Entscheidungsgrenze,
- generativ, d. h. ein Top-down-Ansatz, der auf der Wahrscheinlichkeitsverteilung der Daten basiert,
- hybrid, d. h. eine Kombination der beiden vorgenannten Ansätze.

Ein umfassender Überblick über die Methoden findet sich in [2]. Neben den vielversprechenden Ergebnissen der Methoden zur Entfernung des Rauschens aus Niedrigdosis-CT-Bildern werden inzwischen Modelle eingeführt, die effizienter als früher verwendet werden.

In einer Studie wurde ein neuartiges Modell zum Entrauschen von Niedrigdosis-CT-Bildern vorgeschlagen, das eine Verfeinerung von Strukturen und eine dynamische Faltung auf mehreren Skalen nutzt, um die Einschränkungen früherer DL-basierter Entrauschungsmethoden zu umgehen [4]. Zu diesen Einschränkungen gehören die unvollständige Nutzung hierarchischer Merkmale und die Tatsache, dass sich Standard-Faltungskerne nicht dynamisch an Änderungen der Eingaben anpassen. Das vorgestellte Modell besteht zum einen aus einem Netzwerk, welches aus den Bilddaten basierend auf Ähnlichkeiten oder Verbindungen, die es erkennt, verschiedene Merkmale extrahiert, und zum anderen einem dynamischen *Wahrnehmungsnetzwerk*, welches schwache Signale bzw. Kontraste verbessern soll. Die gefundenen Merkmale beider Netzwerke werden abhängig vom Ort fusioniert, um empfindliche Gewebe in CT-Bildern besser zu erfassen. Zudem wird eine Rauschunterdrückung durchgeführt. Die Methode zeigte im Vergleich zu einigen der gängigen Niedrigdosis-CT-Entrauschungsalgorithmen signifikante Ergebnisse in Bezug auf das Spitzen-Signal-Rausch-Verhältnis (PSNR) und die strukturelle Ähnlichkeit (SSIM). Die Autoren kamen zu dem Schluss, dass die Methode in der Lage ist, ein schnelles Entrauschen durchzuführen und dabei die Details zu erhalten, die bei der Diagnose in der Niedrigdosis-CT-Bildgebung hilfreich sein können.

In einer anderen Studie wurde ein progressives zyklisches neuronales Faltungsnetzwerk („progressive cyclic convolutional neural network“, PCCNN) für den Einsatz in Niedrigdosis-CT-Bildern vorgestellt [5]. Das Modell wurde entwickelt, um Rauschen aus CT-Bildern zu entfernen und gleichzeitig den Speicherbedarf zu minimieren, wodurch es sich besser für den klinischen Einsatz eignet. Dieser auf Deep Learning (DL) basierende Ansatz benötigt vergleichsweise wenig Daten für das Training. Das System enthält ein progressives Modul, welches das Rauschen durch mehrstufige Wavelet-Transformationen effektiv entfernt, ohne dabei hochfrequente Komponenten wie Kanten und Details zu opfern. Im Vergleich zu den sieben in der vorangegangenen Studie erwähnten Algorithmen zum Entrauschen von Niedrigdosis-CT-Bildern zeigte das Framework eine ausgezeichnete Entrauschungsleistung, indem es das PSNR von 29,622 auf 30,671 und den SSIM von 0,8544 auf 0,9199 erhöhte. Die Entrauschungsergebnisse des PCCNN-Verfahrens sind sehr gut und statistisch signifikant erhöht. Bei der qualitativen Bewertung ergab die PCCNN-Methode Bilder mit höherer Auflösung, quasi vollständiger Detailerhaltung und einer strukturellen Gesamttextur, die der einer CT mit normaler Dosis näherkommt, ohne zusätzliche Unschärfe oder Artefakte zu erzeugen. Darüber hinaus erzielte die Methode ein ausgewogenes Ergebnis bei der Rauschunterdrückung, der Beibehaltung des Kontrasts und der Unterscheidung von Läsionen. Eine weitere interessante Studie in diesem Bereich befasste sich mit der Ausnutzung der Verteilungseigenschaften der Niedrigdosis-Bildinformationen, um die Rauschunterdrückung zu verbessern und gleichzeitig detaillierte Informationen zu erhalten [6]. Der zu diesem Zweck entwickelte Swin-Transformer (STEDNet) zeigte im Vergleich zu bestehenden Entrauschungsverfahren eine deutlich bessere Leistung. In Bezug auf den RMSE wurden Verbesserungen von 18,82 %, 15,15 %, 2,25 % bzw. 1,10 % festgestellt, während der PSNR um 9,53 %, 7,33 %, 2,65 % bzw. 3,69 % verbessert wurde. Die Methode könnte das Problem des erhöhten Rauschens und der Artefakte in CT-Bildern lösen und gleichzeitig die Integrität der Gewebestruktur und der pathologischen Informationen bewahren.

Infolge umfangreicher Forschungsarbeiten zum Einsatz KI-basierter Methoden bei der Rekonstruktion und dem Entrauschen von CT-Bildern wurden kommerzielle, intelligente Lösungen für die CT-Bildrekonstruktion eingeführt [7]. Dazu gehören TrueFidelity (General Electric Healthcare, Chicago, USA), AiCE (Canon Medical Systems, Otawara, Japan), Precise Image (Philips Healthcare, Amsterdam, Niederlande), PixelShine (AlgoMedica, Kalifornien, USA) und ClariCTAI (ClariPi, Seoul, Südkorea), die zu einer besseren Qualität der CT-Bildgebung unter dosisarmen Aufnahmebedingungen beitragen. Diese Entwicklungen unterstreichen die wachsende Bedeutung der KI in der medizinischen Bildgebung und ihr Potenzial, die CT-Bildrekonstruktion zu revolutionieren und die Strahlendosis der Patienten zu verringern bzw. die diagnostische Genauigkeit in der klinischen Praxis verbessern.

## Künstliche Intelligenz in der Niedrigdosis-PET

**Figure 3:**
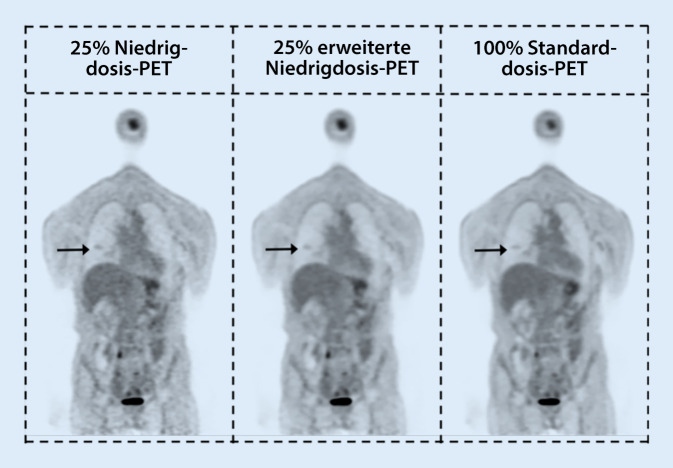

Auch für die nuklearmedizinische Bildgebung spielt der Einsatz der KI eine zunehmende Rolle. In Abb. 3 wird eine beispielhafte Anwendung eines KI-Algorithmus zum Entrauschen von Niedrigdosis-PET-Daten vorgestellt. In [8] wird ein Überblick über die Anwendung von KI in der diagnostischen Nuklearmedizin gegeben. Um Bilder mit normaler Countrate aus Bildern mit geringer Countrate zu berechnen, haben Forscher verschiedene KI-Techniken eingesetzt, vor allem die allgemein verwendeten Ansätze des „convolutional neural network“ (CNN) und des „generative adversarial network“ (GAN) nutzen. Das Hauptziel dieser Studien, die sich in Architektur und Implementierung unterscheiden, bestand darin, qualitativ hochwertige PET-Bilder zu erhalten, indem entweder eine geringere als die Standardmenge an Radioaktivität oder eine niedrigere als die für qualitativ hochwertige PET-Bilder erforderliche Countrate verwendet wird.

In einer Studie, die den Einsatz eines 3D-CNN für die Rauschunterdrückung von PET-Bildern untersuchte [10], wurden PET-Scans von Lungenkrebspatienten in unterschiedlichem Maße verrauscht und ein Modell zur Rauschunterdrückung mit Full-Count-Rekonstruktionen als Referenz trainiert. Die Bewertungen der Ärzte zeigten, dass CNN-entrauschte Bilder bei allen Rauschpegeln mindestens genauso gut oder besser als Gauß-geglättete Bilder waren, wobei die signifikantesten Verbesserungen bei den Bildsätzen mit der niedrigsten Anzahl zu verzeichnen waren. CNN-entrauschte Bilder zeigten eine Wiederherstellung des Läsionskontrasts von 60 % bzw. 90 % bei 1 bzw. 20 Mio. Counts. Während die Wiederherstellung des Läsionskontrasts bei verrauschten Daten geringer ausfiel, boten CNN-verrauschte Bilder eine bessere Erkennbarkeit von Läsionen bei niedrigen Zählwerten, mit einer durchschnittlichen richtig-positiven Erkennungsrate von 40 % bei 1 Mio. echter Counts im Vergleich zu 30 % bei geglätteten Bildern. Obwohl die Technik nur begrenzte Vorteile für die Erkennungsleistung bei Bildern mit in der Klinik üblichen Countlevels hatte, konnte sie die Bildqualität bei sehr verrauschten Bildern erheblich verbessern und bot eine gewisse Verbesserung bei allen Rauschwerten. Eine große Herausforderung beim Entrauschen von PET-Bildern durch End-to-End-Mapping zwischen Bildern mit niedriger und normaler Dosis ist der Verlust von Strukturinformationen, was die Trennung von Rauschen und Signalen erschwert [11]. Dieses Problem wurde durch die Ergänzung von PET-Bildern mit anatomischen Bildern aus der MRT angegangen. In einer Studie wurde ein ortsadaptives, multimodales generatives adversariales Netzwerkmodell (GAN, die deutsche Übersetzung wäre: erzeugende gegnerische Netzwerke) eingeführt, das PET-Bilder mit niedriger Dosis und T1-gewichtete MRT-Scans für eine verbesserte PET-Bildsynthese kombinierte [12]. Die vorgeschlagene Methode erzielte einen höheren PSNR-Wert als die L‑PET-Variante (Niedrigdosis-PET) mit nur der PET-Modalität, was darauf hindeutet, dass anatomische Informationen aus der MRT wichtige Hinweise für die PET-Bildsynthese liefern. Neben den Bemühungen, mit Hilfe von KI-Algorithmen qualitativ hochwertige Bilder aus Niedrigdosis-PET-Daten zu gewinnen, konzentriert sich ein Teil der Studien auf die Verringerung der Scanzeit. Während der erste Ansatz den Strahlenschutz der Patienten verbessert, zielt der zweite Ansatz darauf ab, für die Patienten die Untersuchungsbedingungen zu verbessern [11]. Ein interessantes Ergebnis ist eine Studie, die darauf abzielte, aus Niedrigdosis-PET-Bildern mit Hilfe von T1-gewichteten MRT-Bildern unter Verwendung einer DL-Architektur Normaldosis-PET-Bilder zu erzeugen [13]. Das vorgeschlagene Modell ordnete die Eingaben direkt der Ausgabe zu, ohne dass die Daten über die Optimierung in der Trainingsphase hinaus verarbeitet wurden. Die Methode führte ein einziges Feed-Forward-Verfahren durch, um das Schätzungsergebnis zu erhalten, was zu einer schnellen Schätzung der PET führte. Tests des trainierten Modells mit echten menschlichen Gehirnbildern haben gezeigt, dass die Methode 500-mal schneller ist als ähnliche Ansätze.

Die umfangreichen Studien in diesem Bereich und die faszinierenden Ergebnisse zeigen, dass die KI das Potenzial hat, die Rekonstruktion von PET-Bildern und die Entrauschungstechniken zu revolutionieren. Durch die erfolgreiche Implementierung von KI-basierten Methoden für die PET-Bildgebung im Niedrigdosis- und Low-Count-Bereich scheint diese Technologie das Potenzial zu haben, bildgebende Verfahren sowohl für Patienten als auch für das Personal sicherer und komfortabler als bisher zu machen und gleichzeitig die diagnostische Genauigkeit zu verbessern.

## Künstliche Intelligenz in der Niedrigdosis-SPECT

Auch bei der SPECT sind Patienten ionisierender Strahlung ausgesetzt. Die Verringerung der für ein Verfahren benötigten Radioaktivität und die Verbesserung der Bildqualität, insbesondere im Zusammenhang mit kardialen Anwendungen, bei denen die Bilder oft verrauscht sind, waren schon immer ein Ziel bei diesem Bildgebungsverfahren. Auch hier haben KI-basierte Entrauschungsmethoden neue Möglichkeiten eröffnet.

**Figure 4:**
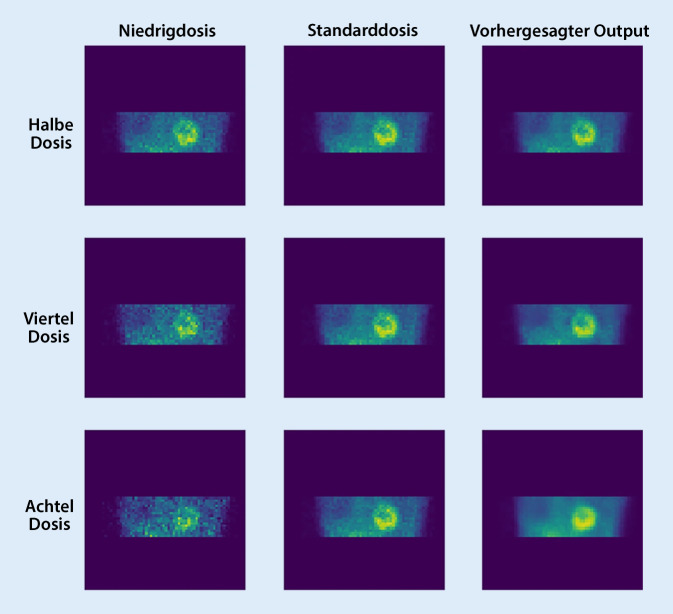

In einer Studie wurde der potenzielle Nutzen eines CNN-Modells zur Unterdrückung von Rauschen in der SPECT-Myokardperfusionsbildgebung im Niedrigdosisbereich untersucht [14]. Die Normaldosis- und Niedrigdosis-Bildpaare von 1052 Probanden wurden zum Trainieren eines neuronalen Netzes verwendet. Die beiden gängigen Rekonstruktionsmethoden, die gefilterte Rückprojektion („filtered back projection“, FBP) und das iterative Ordered-subset-expectation-maximisation-Rekonstruktionsverfahren (OSEM, deutsch etwa: Erwartungswertmaximierung verwendende iterative Rekonstruktion basierend auf geordneten Teildatensätzen), wurden bei 4 Dosisstufen, bei der Hälfte, einem Viertel, einem Achtel und einem Sechzehntel der vollen Dosis, getestet. Die Ergebnisse der Studie zeigten, dass der DL-Ansatz eine signifikante Rauschreduzierung und eine verbesserte diagnostische Genauigkeit von Daten mit niedriger Dosis erzielte. Bei der halben Dosis erreichte die DL eine Fläche unter der ROC(„receiver operator characteristics“)-Kurve (AUC, „area under the curve“) von 0,799, die fast identisch mit der AUC von 0,801 bei der OSEM-Volldosis war. Selbst bei einer Dosis von einem Achtel erreichte DL eine AUC von 0,770 für OSEM, die höher war als die AUC von 0,755 für FBP in voller Dosierung. Die Ergebnisse deuten darauf hin, dass die DL-Entrauschung im Vergleich zur konventionellen Rekonstruktionsfilterung eine zusätzliche Dosisreduktion ohne Beeinträchtigung der diagnostischen Genauigkeit bei SPECT-Maximumintensitätsprojektion (MPI) ermöglicht. In einer anderen, ähnlichen Studie wurde ein GAN-Modell entwickelt, um aus Niedrigdosisbildern mit verschiedenen Radioaktivitätswerten Normaldosisbilder von SPECT-MPI zu erzeugen [15]. Für die Studie wurden klinische Daten von 345 Patienten verwendet. Bei einer Reduktion der Dosis auf die Hälfte, ein Viertel und ein Achtel der Standarddosis lag der Prozentsatz der klinisch akzeptablen Bilder bei 100 %, 80 % bzw. 11 %. Mit anderen Worten: Das vorgeschlagene Netzwerk unterdrückte wirksam das Rauschen und lieferte bei halber und viertel Dosis vergleichbare, vorhergesagte Bilder der Standarddosis. Abb. 4 zeigt ein Bild aus der Studie, aus dem deutlich hervorgeht, wie die Bilder bei der Hälfte, einem Viertel und einem Achtel der Standarddosis durch KI-Algorithmen verbessert wurden. In einer anderen Studie wurde die Wirksamkeit eines gekoppelten U‑Netzes mit herkömmlichen Nachfilterungsmethoden zur Entrauschung in der SPECT-Myokardperfusionsbildgebung und zur Verbesserung der Erkennbarkeit von Perfusionsdefekten verglichen [16]. In der Studie wurde eine gekoppelte U‑Netz-Struktur mit einem Rauschen-zu-Rauschen-Trainingsansatz verwendet, um den Mangel an „ground truth“ in klinischen Studien zu überwinden. Der vorgeschlagene Ansatz wurde an einem Satz von 895 klinischen Studien getestet, wobei der iterative OSEM-Algorithmus mit 3D-Gauß-Nachfilterung zur Rekonstruktion der Bilder verwendet wurde. Die Ergebnisse zeigen, dass der Deep-Learning-Ansatz die Erkennungsleistung von Perfusionsdefekten auf allen Kontraststufen im Vergleich zur OSEM-Methode mit Gaußscher Nachfilterung deutlich verbessert und eine zusätzliche Rauschunterdrückung in SPECT-MPI-Bildern bewirkt, was ihn zu einem vielversprechenden Ansatz für die Verbesserung der Perfusionsdefekterkennung macht.

Fasst man das Wesentliche zum Entrauschen von SPECT-Bildern mit intelligenten Methoden zusammen, wird deutlich, dass der Einsatz von KI-basierten Methoden in der SPECT-Bildgebung vielversprechend ist, um die Bildqualität bei reduzierter Strahlendosis zu verbessern und so eine sicherere SPECT-Praxis zu fördern. Es ist zu erwarten, dass die sich rasch entwickelnde KI-Technologie und die Integration von KI-basierten Bildrekonstruktionsverfahren in SPECT-Bildgebungssysteme alltäglicher werden und sowohl der Patientensicherheit als auch der diagnostischen Genauigkeit zugutekommen.

## Künstliche Intelligenz in der Niedrigdosis-Angiographie

Neben den Fortschritten bei den diagnostischen Bildgebungsverfahren wird die KI auch in der interventionellen Bildgebung einsetzbar sein. Hier ist zu erwarten, dass ihr Einsatz hier besonders sinnvoll ist, weil jeweils viele ähnliche Daten im Zeitverlauf generiert werden; die KI kann besonders gut Informationen zusammenbringen und Vorinformationen sinnvoll integrieren, so dass deutliche Expositionsreduktionen möglich sein sollten. Die Angiographie ist ein häufig verwendetes bildgebendes Verfahren zur Darstellung von Blutgefäßen mit Hilfe ionisierender Strahlung. Es gibt interessante Berichte über den Einsatz kommerzieller KI-gestützter Verfahren, mit denen die Strahlendosis für die Patienten erfolgreich reduziert werden kann, ohne dass die Qualität der Bilder beeinträchtigt wird.

In einer Studie wurden der diagnostische Wert und die Fähigkeit zur Dosisreduktion einer KI-basierten 3D-Angiographie (3DA) bei der Visualisierung von intrakraniellen Arterienstenosen (IAS) untersucht und mit der standardmäßigen digitalen 3D-Subtraktionsangiographie (3D-DSA) verglichen [17]. Die IAS-Datensätze wurden mit konventioneller und Prototyp-Software nachbearbeitet und anschließend von erfahrenen Neuroradiologen hinsichtlich Bildqualität, Gefäßdurchmesser, Gefäßgeometrie-Index und spezifischer IAS-Parameter wie Lage und visuelle Einstufung bewertet. Die Ergebnisse zeigten, dass 20 angiographische 3D-Volumina erfolgreich rekonstruiert wurden und die Bildqualität von 3DA und 3D-DSA gleichwertig war. Die Bewertung der Gefäßgeometrie in 3DA-Datensätzen unterschied sich nicht signifikant von 3D-DSA. Die Autoren kamen zu dem Schluss, dass die KI-basierte 3DA ein zuverlässiger Algorithmus für die Visualisierung von IAS ist, der vergleichbare Ergebnisse wie 3D-DSA liefert und gleichzeitig eine deutlich geringere Strahlenbelastung des Patienten ermöglicht. In einer anderen Studie wurde die Bildqualität von Niederspannungs-Koronar-CT-Angiographie-Bildern, die mit kommerziellen DL-Bildrekonstruktionsverfahren rekonstruiert wurden, bewertet und mit der von FBP und adaptiver statistischer iterativer Rekonstruktion‑V (ASiR-V50 %) verglichen [18]. Insgesamt wurden 80 Patienten in 2 Gruppen gescannt, entweder bei 70 kVp oder 80 kVp, je nach Body-Mass-Index (BMI). Die Ergebnisse der Studie zeigten, dass die KI-basierten Ansätze die FBP- und AsiR-V50 %-Rekonstruktionsmethoden übertrafen und das geringste Rauschen, das höchste SNR, das beste Kontrast-Rausch-Verhältnis (CNR) und den geringsten Kantenanstiegsabstand lieferten.

**Figure 5:**
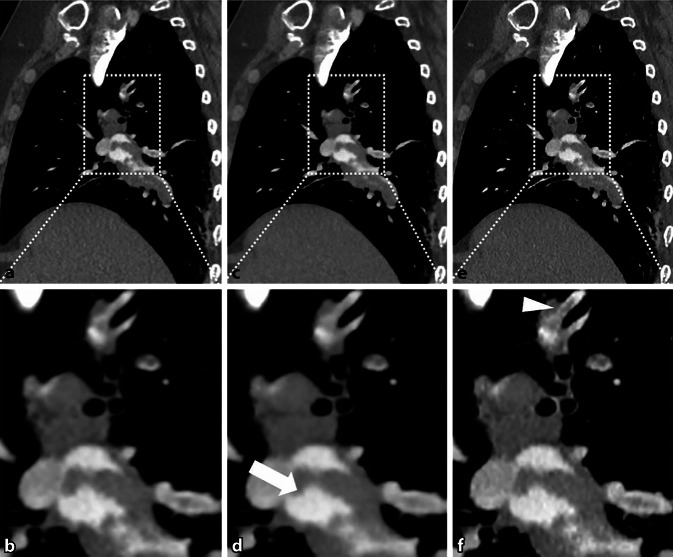

In einer anderen Studie wurden die Bildqualität und die Strahlendosis bei der computertomographischen Lungenangiographie mit einem DL-Rekonstruktionsalgorithmus im Vergleich zu einem hybrid-iterativen Rekonstruktionsverfahren (IR) bewertet [19]. Die Studie umfasste 93 Patienten, bei denen der Verdacht auf eine Lungenembolie (PE) bestand. Die Analyse ergab, dass die DL-Rekonstruktionsbilder im Vergleich zu den Hybrid-IR-Bildern ein deutlich geringeres Rauschen und ein besseres SNR und CNR aufwiesen. Abb. 5 zeigt die mit den beiden Verfahren rekonstruierten Bilder. Es wurde kein signifikanter Unterschied in der Diagnosesicherheit für PE zwischen den Techniken festgestellt.

Studien zur Koronar-CT-Angiographie und zur Lungenangiografie haben gezeigt, dass die auf Deep Learning basierende Bildrekonstruktion die Strahlenexposition reduzieren kann, ohne die diagnostische Präzision oder Bildqualität zu beeinträchtigen.

## Künstliche Intelligenz in der Niedrigdosis-Fluoroskopie

**Figure 6:**
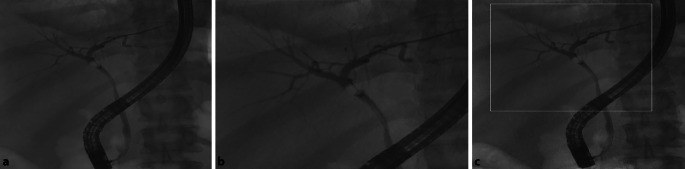

Die Durchleuchtung ist ein weiteres bildgebendes Verfahren, das im Operationssaal bewegte Bilder der inneren Struktur des Körpers liefert. Diese Bildgebungsmethode dient u. a. der Implantation von Kathetern, Stents oder anderen medizinischen Geräten. Aufgrund der kontinuierlichen und Echtzeitbelichtung sind Patienten und medizinisches Personal bei der Fluoroskopie in der Regel einer erhöhten ionisierenden Strahlung ausgesetzt. Dieser Bildgebungsbereich kann erheblich von der KI-Technologie profitieren, um die Sicherheit der Patienten zu erhöhen. In einer Studie wurde gezeigt, dass KI-Algorithmen die Belastung durch ionisierende Strahlung sowohl für Patienten als auch für medizinisches Fachpersonal bei durchleuchtungsgeführten endoskopischen Verfahren wirksam verringern können [20]. In dieser Studie wurde die konventionelle Fluoroskopie mit der KI-integrierten Fluoroskopie verglichen, bei der eine ultraschnelle Kollimation eingesetzt wurde, um die Strahlenexposition auf den interessierenden Bereich zu beschränken. Um den Vergleich fair zu halten, wurden die demografischen Daten, der BMI, die Art des Verfahrens und die Verfahrens- bzw. Durchleuchtungsdauer nahezu identisch gehalten. Die Ergebnisse der Studie zeigten, dass das KI-gestützte Durchleuchtungssystem die Strahlenbelastung der Patienten im Vergleich zu konventionellen Systemen deutlich reduziert. Darüber hinaus war ein deutlicher Rückgang der Streustrahlung für das Endoskopiepersonal zu verzeichnen. Ein Vergleich der konventionellen Fluoroskopie mit der KI-integrierten Fluoroskopie ist in Abb. 6 dargestellt. Das System überlagert die Echtzeitbilder der ROI mit einem Bild des gesamten Sichtfelds, das einige Bilder zuvor aufgenommen wird. Die Studie zeigte das vielversprechende Potenzial der KI-Technologie bei der Wiederherstellung verrauschter Niedrigdosis-Durchleuchtungsbilder, die dem Arzt klinisch wertvolle Informationen liefern und gleichzeitig den Strahlenschutz der Patienten verbessern.

Wenn man die erfolgreiche KI-Forschung und -Anwendung in verschiedenen Bereichen der medizinischen Bildgebung zusammenfasst, wird deutlich, dass die KI-Technologie verrauschte Bilder mit geringer Dosis in klinisch wertvolle Bilder umwandeln und den Strahlenschutz für Patienten und Personal verbessern kann. Dieser potenzielle Fortschritt in der medizinischen Bildgebung unterstreicht die Bedeutung kontinuierlicher Forschung und Entwicklung auf dem Gebiet der KI und ebnet den Weg für weitere Verbesserungen in der Patientenversorgung und im Strahlenschutz. Allerdings gibt es auch viele potenzielle Schwierigkeiten und Probleme bei der Anwendung von KI-basierten Verfahren in der medizinischen Bildgebung, insbesondere mit Bezug zu Verfahren, die auf der Verwendung ionisierender Strahlung beruhen.

## Herausforderungen

Der Einsatz von KI und DL in der medizinischen Bildgebung hat das Potenzial, die Strahlendosis zu verringern, allerdings mit gewissen Vorbehalten und unter bestimmten Voraussetzungen. Sind diese Voraussetzungen nicht erfüllt, können im Sinne des Strahlenschutzes erhebliche Probleme entstehen: Da medizinische Bilder, die mit Methoden der KI rekonstruiert und/oder entrauscht wurden, immer sehr gut aussehen, scheinbar feine Details enthalten und ein sehr gutes Signal-zu-Rausch-Verhältnis aufweisen, ist für den betrachtenden Mediziner nicht nachvollziehbar, ob alle (relevanten) Details korrekt abgebildet sind oder ob einige verschwunden oder hinzugekommen oder in relevanter Weise von der medizinischen bzw. anatomischen Realität abweichen. Dies ist kritisch im Sinne der Diagnoseerstellung, sowohl weil eine falsche Sicherheit erzeugt wird, als auch weil Fehldiagnosen und zwar im Sinne von falsch-positiven wie falsch-negativen Befunden entstehen können. Um dies zu verhindern, müssen grundsätzlich bestimmte Rahmenbedingungen und Voraussetzungen gegeben sein:

- Trainingsdaten, Test- und Validierungsdaten müssen voneinander unabhängig sein.
- Es müssen ausreichend große Trainingsdatensätze, die vorgegebene Qualitätskriterien erfüllen, vorliegen.
- Im Fall von überwachten Lernmodellen muss die grundlegende Wahrheit („ground truth“) möglichst exakt bekannt sein.
- Es muss realisiert werden, dass die durch KI erzeugten Zusatzinformationen (geringeres Rauschen, Detailschärfe etc.) nur sicher sind, sofern sie durch physikalische Messgrößen erzeugt werden (z. B. aus Voruntersuchungen etc.) oder sog. Sparsitybedingungen ausgenutzt werden können (d. h. dass in der Aufnahme eines vollständigen Datensatzes Daten vorhanden sind, die keine eigene Information enthalten). Die Informationsmatrix ist also dünn besetzt. Kann man die nicht relevanten Datenpunkte bestimmen und damit die benötigten Daten in einer Art und Weise reduzieren, die die Aufnahme weniger Daten erlaubt, ist eine physikalisch korrekte Rekonstruktion generell möglich. Je mehr Daten ohne diese Bedingungen erzeugt werden, umso größer wird die Wahrscheinlichkeit für Fehlinformationen oder unterdrückte Informationen.
- Eine Qualitätssicherung ist erforderlich. Diese muss komplett neue Wege z. B. von sich ändernden Phantomen verfolgen, da das KI-System bei Wiedererkennung von Phantomen diese natürlich ideal darstellen würde.

Ein mögliches Problem besteht wie angegeben im Mangel an ausreichenden Daten, die zum Trainieren der datenintensiven DL-Modelle benötigt werden. DL-Modelle erfordern die Erstellung umfangreicher Datenbanken oder die Erforschung alternativer Lernmethoden, einschließlich unbeaufsichtigtem, selbstüberwachtem oder schwach überwachtem Lernen.

Abweichungen zwischen verschiedenen Standorten, Scannertypen und Tracern, im Fall von PET- und SPECT-Studien, können die Leistung des Modells und seine Verallgemeinerbarkeit beeinträchtigen. Die Entwicklung von Modellen, die sich an verschiedene Scanner, Organe, Bildgebungsprotokolle, Rauschpegel und Dimensionen anpassen können, ist für ihre praktische Anwendung unerlässlich. Die Optimierung von verwendeten Parametern und Netzwerkarchitekturen ist entscheidend für die Erzielung genauer Ergebnisse. Die Entwicklung von Mechanismen zur optimalen Initialisierung dieser Faktoren ist daher ein wichtiges Forschungsgebiet.

Mangelnde Transparenz und Interpretierbarkeit, die oft mit DL-Modellen verbunden sind, erschweren die Akzeptanz dieser Modelle in der medizinischen Gemeinschaft. Die Verwendung von erklärbaren KI-Modellen kann helfen, dieses Problem zu lösen. Allerdings wird das Einsatzmöglichkeiten beschränken und ggf. auch nicht alles erklärbar machen.

Ethische und rechtliche Erwägungen sollten sorgfältig berücksichtigt werden, um den Datenschutz zu gewährleisten, die Wünsche der Patienten zu respektieren und ihr Einverständnis zu der Anwendung der Verfahren zu erhalten, um eine verantwortungsvolle Einführung von KI in der medizinischen Bildgebung sicherzustellen.

Die Implementierung von KI-basierten Lösungen im klinischen Umfeld erfordert eine nahtlose Integration in bestehende klinische Arbeitsabläufe und Systeme, was aufgrund unterschiedlicher Praktiken, Protokolle und Infrastrukturen im Gesundheitswesen eine Herausforderung darstellen kann.

Eine standardisierte Validierung und Qualitätskontrolle von Methoden ist entscheidend, um die Leistung und Robustheit von KI-Modellen in der medizinischen Bildgebung zu bewerten und den Übergang der Modelle vom Labor zum Krankenbett zu ermöglichen. Die Gewährleistung der Robustheit von KI-Modellen gegenüber Angriffen von außen und potenziellen Sicherheitsverletzungen ist für die Aufrechterhaltung der Integrität medizinischer Bildgebungssysteme entscheidend. Nicht zuletzt geht es darum, Methoden zu entwickeln, die eine nahtlose Kommunikation zwischen KI-Modellen und Medizinern ermöglichen. Dies ist der Schlüssel zum Erreichen des ultimativen Ziels der Entwicklung und des Einsatzes von KI-Modellen in der Radiologie, d. h. der Ergänzung des Fachwissens von Ärzten durch die Stärken von KI-Modellen, um den Strahlenschutz der Patienten und die Behandlungsergebnisse zu verbessern.

Es sind also weitere Forschungs- und Entwicklungsarbeiten erforderlich, um das Potenzial von KI und DL in der medizinischen Bildgebung zur Dosisreduzierung voll auszuschöpfen und die klinische Anwendbarkeit zu gewährleisten.

## Fazit für die Praxis

- Vielversprechende Berichte über den Einsatz von künstlicher Intelligenz (KI) in verschiedenen Anwendungen der medizinischen Bildgebung haben gezeigt, dass sie das Potenzial hat, die Exposition der Patienten (und des beteiligten Personals) mit ionisierender Strahlung zu verringern.
- Dieses Versprechen liegt in der Fähigkeit moderner KI-Algorithmen, Bilder zu entrauschen, die mit geringer Dosis aufgenommen wurden, und so die Qualität der Bilder auf ein klinisch akzeptables Niveau zu heben.
- Damit die KI-Technologie in die medizinische Bildgebungsroutine integriert werden kann, müssen die Herausforderungen überwunden werden, die sich bei der Implementierung dieser Technologie im klinischen Umfeld stellen; dazu gehören u. a. unzureichende Daten, die Verallgemeinerbarkeit von Modellen, Optimierung, Transparenz, ethische und rechtliche Erwägungen sowie die Robustheit gegenüber computertechnischen bzw. Software-Angriffen.
- Die Bewältigung dieser Herausforderungen durch fortgesetzte Forschung und Entwicklung wird entscheidend sein, um das Potenzial der KI in der medizinischen Bildgebung zur Dosisreduzierung voll auszuschöpfen und letztlich eine sicherere medizinische Bildgebungskultur zu fördern.
